# Bioactive Compounds in Different Buckwheat Species

**DOI:** 10.3390/plants10050961

**Published:** 2021-05-12

**Authors:** Grażyna Podolska, Elżbieta Gujska, Joanna Klepacka, Edyta Aleksandrowicz

**Affiliations:** 1 Department of Cereals Crop Production, Institute of Soil Science and Plant Cultivation-State Research Institute, Czartoryskich 8 Str, 24-100 Puławy, Poland; grazyna.podolska@iung.pulawy.pl; 2 Department of Commodity Science and Food Analysis, University of Warmia and Mazury in Olsztyn, Plac Cieszynski 1, 10-719 Olsztyn, Poland; elka@uwm.edu.pl (E.G.); klepak@uwm.edu.pl (J.K.)

**Keywords:** *Fagopyrum esculentum*, *Fagopyrum tataricum*, cultivars, phenolic acid, rutin, minerals, protein

## Abstract

The accumulation of valuable nutrients in cereal grains depends on a number of factors, including species, cultivars, and environment conditions. The aim of this study was to compare protein, some polyphenols and rutin content, as well as mineral composition in *Fagopyrum tataricum* and *Fagopyrum esculentum* genotypes growing in Polish conditions. A field experiment was conducted on pseudopodsolic soil in 2017–2018 at the Experimental Station in Osiny (51°35′, 21°55′), following randomized complete block method with three replications. Two cultivars of *Fagophyrum esculentum* (Kora and Panda), two cultivars of *Fagopyrum tataricum* (LIT1 and 63181) and two forms of *Fagopyrum esculentum* (Red Corolla and Green Corolla) were used in this experiment. We found differences in the tested compounds (protein, phenolic acids, rutin, and mineral composition) between cultivars and genotypes. Total phenolic acid and rutin contents were higher in the *Fagopyrum tataricum* compared to *Fagopyrum esculentum*. Ferulic and coumaric acids were prominent in the Kora and Panda cultivars, however vanillic and syringic acids accumulated more in Green Corolla and Red Corolla. The common buckwheat seeds contained more Cu, Mn, and Mg and less Ca than tartary buckwheat. Moreover *Fagopytum esculentum* genotype contains more protein compared to *Fagopyrum tataricum*.

## 1. Introduction

Common buckwheat (*Fagopyrum esculentum*) and tartary buckwheat (*Fagopyrum tataricum*) belong to the genus *Fagopyrum* of the family *Polygonaceae*, and they are among the main buckwheat species cultivated worldwide [1]. Tartary buckwheat is grown and used in the mountainous regions of southwest China, northern India, Bhutan, and Nepal. Common buckwheat is grown mainly in Europe (Russian Federation, Ukraine, Poland) [2,3]. Buckwheat was brought to Poland by the Mongols in the 13th century; however, the first records of buckwheat as a cultivated plant in Poland date back to the 16th century. Records show that, since then, common and tartary buckwheat have been grown across Europe, including the countries of Russia, Ukraine, Tajikistan, Kazakhstan, Lithuania, Estonia, Belarus, Moldova, Poland, Yugoslavia, Croatia, Slovenia, Austria, France, and Italy. During the 20th century, tartary buckwheat virtually disappeared as a crop in Europe. However, Bonafaccia [4] did report that until the 1980s small quantities of tartary buckwheat were still being grown in parts of central Europe, particularly in the alpine region. Nowadays, in Poland, tartary buckwheat occurs only as a weed, contaminating the common buckwheat fields. It seems, however, this might soon change, as in recent years interest in alternative food sources is growing and tartary buckwheat has been included in the search for novel valuable food for human consumption [5]. Impressed by its high nutritional value, researchers have been promoting the revival of this species as a “functional food.” The beneficial effects of buckwheat result from its high levels of polyphenol compounds such as flavonoids, and phenolic acids which exhibit antioxidant activity [6,7,8]. Studies of buckwheat antioxidants indicated that environmental and weather conditions, growing season, species, cultivar influenced the phenolics, flavonoids, and rutin content of common and tartary buckwheat seed [7,9,10]. The mineral content varied in the range from 2.0 to 2.5%. Buckwheat grains are an important source of microelements, such as: Zn, Cu, Mn, Se [11], and macroelements: K, Na, Ca, and Mg [12,13]. Most of all, the content of minerals is influenced by cultivars and species. The literature sources show differences in microelements and trace elements between *Fagopyrum esculentum* and *Fagopyrum tataricum*. Compared to tartary buckwheat, common buckwheat grains contain larger concentrations of S, Ca, Cu, and Mo [14]. The higher concentrations of Se, Zn, Fe, Co, and Ni were found in the grains of tartary buckwheat [4]. Although the genotype plays a major role in shaping the grain quality traits, the environmental conditions (especially climatic conditions) and the applied agricultural techniques matter as well. A protein concentration of 12–19% is generally observed in buckwheat grains. This content however is highly dependent on species, various environmental factors, as well as crop management practices [15,16]. When grains are analyzed qualitatively, tartary buckwheat achieves a higher concentration of proteins and amino acids compared to the common buckwheat [17,18]. Interestingly, buckwheat proteins possess the highest amino acid score of 100 when compared to other plant sources. Additionally, buckwheat has a very high biological value due to its high lysine content compared to other cereals [19].

Therefore, of high importance is the evaluation of the level of bioactive compounds in different genotypes growing in the same environmental conditions. As a result, the research hypothesis states that in Polish climatic and soil conditions, tartary buckwheat produces large amounts of health-promoting compounds, whereas the amount of these compounds depends on the genotype. We also hypothesize that buckwheat genotypes adapted to the climatic conditions of Poland differ in the amount of bioactive ingredients. The aim of this study was to compare protein, polyphenol, and rutin content, as well as mineral composition in selected buckwheat genotypes growing in Polish conditions.

## 2. Results

### 2.1. Phenolic Acids

There were significant differences in the total phenolic acid content of the tested genotypes of buckwheat. The highest amount of phenolic acid was found in tartary buckwheat, follow by Red Corolla, Green Corolla, Panda, and finally Kora cv. The total phenolic acid content in *Fagopyrum tataricum* ranged from 6948.9 mg·kg^−1^ d.w (63,481 cv.) to 7014.8 mg·kg^−1^ d.w (LIT1 cv.). This is 1.7–3-fold higher than in common buckwheat with a range of 2222.2 mg·kg^−1^ d.w (Kora cv.) to 2322.7 mg·kg^−1^ d.w (Green Corolla). However, there was no difference for the total phenolic acid content between varieties of the same species *F. tataricum* LIT 1 cv. and 63481 cv. and between varieties of *F. esculentum* Kora and Panda. Red Corolla genotypes had significantly higher content of total phenolic acid than Kora, Panda, and Green Corolla (Table 1).

The highest amount of ferulic acid was found in Kora cv., whereas the lowest was found in Green Corolla. We also found differences between contents of ferulic acid of cultivars belonging to the same species. The content of ferulic acid was 1.5 times higher in tartary buckwheat 63481 cv. than in LIT 1 cv. The content of ferulic acid in genotypes belonging to *F. esculentum* ranged from 2.266 mg·kg^−1^ d.w (Green Corolla) to 4.000 mg·kg^−1^ d.w (cv. Kora).

Buckwheat belonging to *F. esculentum* contained the biggest amount of coumaric acid compared to *F. tataricum*. The biggest amount (2.6 times higher) was found in Panda grains (39.45), and the lowest was found in *F. tataricum* LTI 1 (15.46). 

The amount of syringic acid differed significantly between the studied genotypes. The content ranged from 38.61 mg·kg^−1^ d.w (LIT 1) to 85.62 mg·kg^−1^ d.w (Red Corolla). The average amount in tartary buckwheat was 1.9-fold lower than in F. esculentum genotypes. The significantly highest content of vanillic acid was found in Green Corolla and Red Corolla genotypes, and the lowest was found in *F. tatarium* cv. (Table 2).

Genotypes significantly varied in the rutin content. The highest amount of rutin was contained in *Fagopyrum tataricum* seeds, followed by Red Corolla, Green Corolla, Panda, and Kora cv. Tartary buckwheat had 11–26-fold higher rutin content compared to Red and Green Corolla; moreover, the rutin content in tartary buckwheat was 26-fold higher than that detected in common buckwheat. The rutin content was 1.6-fold higher in the seeds of LTI 1 cv. than in the ones of 63,481 cv. in *F. tataricum*. However, there were no differences between cultivars of *F. esculentum* (Kora and Panda) (Table 2).

**Figure 1:**
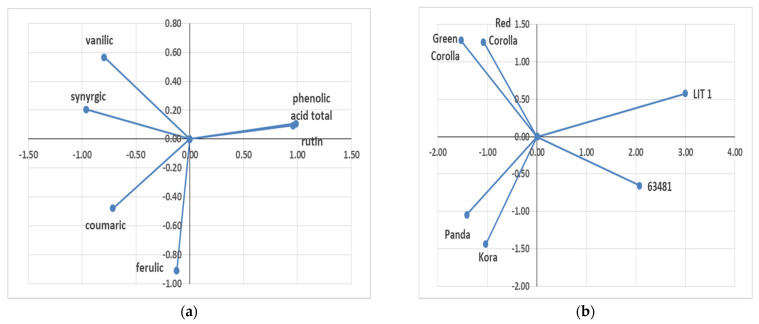
Principal component analysis (PCA) of seed bioactive compounds of *F. esculentum* and *F. tataricum* genotypes (**a**) and individual graph of the tested genotypes (**b**).

A principal component analysis (PCA) was performed in order to identify potential differences between genotypes regarding bioactive compounds of seeds. Axis 1 was mainly explained by, on one side, the total phenolic acid and rutin, and on the other side, vanillic acid, syringic acid, coumaric, and ferulic acid. The analysis of PCA showed interesting interactions between total phenolic acid contents and rutin content. Furthermore, significant interaction between vanillic and synergic acid and between coumaric and ferulic acid (Figure 1a) was detected. The PCA analysis additionally showed the clear separation of bioactive compound contents between the tested genotypes (Figure 1b). The genotypes can be divided in four groups. The first is Lit 1, the second is Green Corolla and Red Corolla, the third is Panda and Kora, and the fourth is 63,481 cv.

### 2.2. Mineral Content

Regarding mineral content, the seeds of *Fagopyrum tataricum* contained significantly less Cu, Mn, and Mg but more Ca than the ones of *Fagopyrum esculentum*. The seeds of Red Corolla and Green Corolla contained more Cu, Mn, Fe, and Zn and less Na than the other tested genotypes. The Red Corolla contained the biggest amount of Mg and P (Table 2).

**Figure 2:**
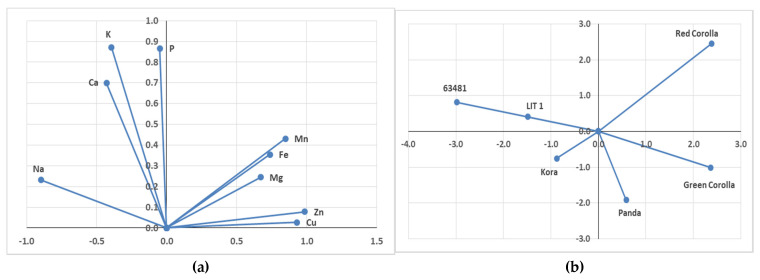
Principal component analysis (PCA) of seed mineral content of *F. esculentum* and *F. tataricum* genotypes (**a**) and individual graph of the tested genotypes (**b**).

A principal component analysis (PCA) was performed in order to identify potential differences between genotypes regarding mineral content of the seeds. Additionally, the PCA showed strong interaction between the Mn, Fe, Mg, Zn, and Cu contents. and interaction between P, K, Ca, and Na contents (Figure 2a). The PCA revealed the clear separation in mineral content between the tested genotypes. The genotypes can be divided into four groups: red corolla, *F. tataricum* cultivars, Kora, fourth Panda, and Green Corolla (Figure 2b).

### 2.3. Protein Content

**Figure 3:**
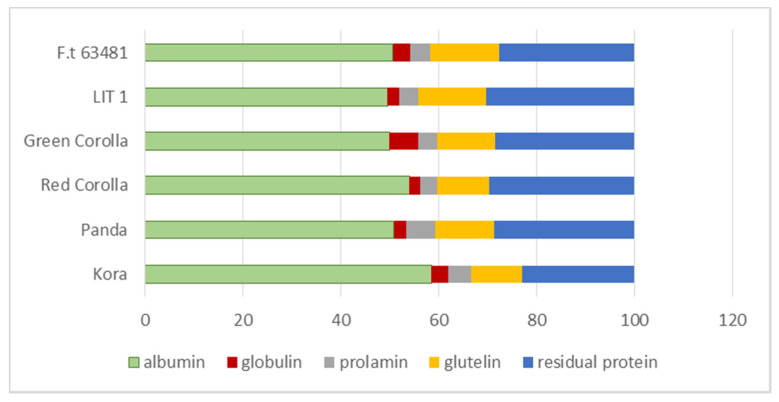
Proportion (%) of protein fractions in buckwheat genotypes (protein = 100%).

The protein content ranged from 11.48 to 14.04% and was 1.18-fold higher in *Fagopyrum esculentum* than in *Fagopyrum tataricum*. The content of albumin fraction ranged from 5.67 to 7.58 (g/100 g d.m.) and was 1.25 times higher in *Fagopyrum esculentum* genotypes than in *Fagopyrum tataricum* cultivars. According to the data in Figure 3, albumins comprised from 49% (LIT 1 cv.) to 58% (Kora cv.) of total protein content. The globulin fraction ranged from 0.28 to 0.83 (g/100 g d.m.). The biggest amount of globulin was found in Green Corolla, the lowest in Panda cv. Red Corolla and LIT 1. The seeds of Kora and Panda cv. contained more prolamin fraction than both *F. tataricum* cv.s. The amount of glutelin was similar in the tested genotypes with the exception of Kora cv., the seeds of which contained significantly less of this protein fraction. This fraction comprised 10% (Kora cv.) to 14% (63481 cv.) of the protein. In varieties of tartary buckwheat it constituted from 13.8 (LIT 1 cv.) to 14.1 percent (63481 cv.) of protein, while in the case of common buckwheat from 10.3 (Kora) to 12.1% (Panda) (Table 3, Figure 3).

**Figure 4:**
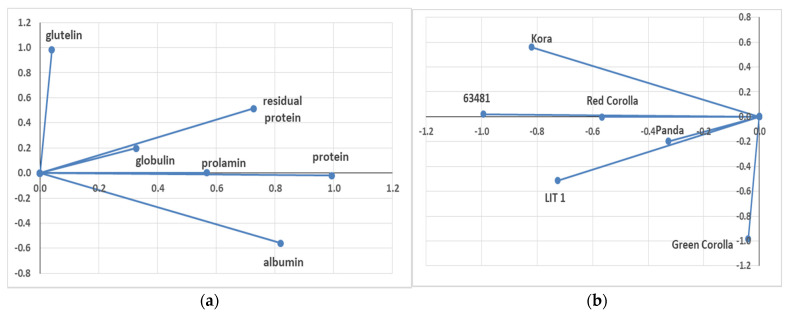
Principal component analysis (PCA) of protein content of *F. esculentum* and *F. tataricum* genotypes (**a**) and individual graph of the tested genotypes (**b**).

A principal component analysis (PCA) was performed in order to identify potential differences between genotypes regarding protein fractions. The PCA showed strong interaction between globulin and residual protein and prolamin and total protein content (Figure 4a). In addition, the PCA showed the differences between the tested genotypes (Figure 4b).

**Figure 5:**
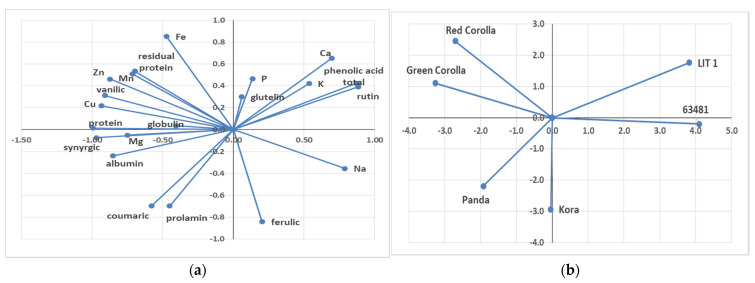
Principal component analysis (PCA) of tested parameters of *F. esculentum* and *F. tataricum* genotypes (**a**) and individual graph of the tested genotypes (**b**).

A principal component analysis (PCA) was performed in order to identify potential differences between species and cultivars regarding bioactive compounds of seeds, mineral content of the seeds, and protein fractions (Figure 5a). The analysis shows strong differences between tested species, as well as similarity between *F. tataricum* cultivars, *F. esculentum* cultivars, and between Red Corolla and Green Corolla (Figure 5b).

## 3. Discussion

Plants produce a very high number of secondary metabolites and about 10,000 molecules belonging to the polyphenols class have been identified to date. One of the main functions of the phenolic derivatives in plants are the structural function and a protective function of photosynthetic systems against excessive radiation. Moreover, their antioxidant and anti-inflammatory properties have attracted a lot of interest in recent decades. The health benefits are due to the plant’s high levels of phenolic compounds [20,21]

In this study, different genotypes and varieties of *Fagopytrym esculentum* and of *Fagopyrum tataricum* were compared under the bioactive compounds (phenolic acids, rutin), mineral, and protein content. To our knowledge, this was the first such study conducted in Polish conditions in the same location under the same crop management practices. In this study, the phenolic acids such as ferulic acids, coumaric acids, syringic acid, and vanillic acid and the flavonoids, such as rutin, were detected in tested genotypes. We found differences between species and varieties, which is confirmed in the literature. Generally speaking, rutin and total phenolic acids contents were much higher in tartary buckwheat cultivars compared to common buckwheat. The average amount of rutin content in both *Fagopyrum tataricum* seeds was 2659 mg·kg^−1^ DW, while in the *Fagopyrum esculentum* genotypes it equaled 167.2 mg·kg^−1^ DW. The big difference in rutin content among species is confirmed in the literature [22]. For example, Aubert et al. [23] prove that the rutin level in *Fagopyrum tataricum* cultivars ranges from 3.44 to 3.79 mg·g^−1^ FW, while in *F. esculentum* cultivar it ranges from 0.1 to 0.02 mg·g^−1^ FW. Guo et al. [7] reported that rutin content in tartary buckwheat ranges from 2077 to 3149 μmol /100 g DW. In our experiment, the rutin level in *F. tataricum* seeds was 15 times higher, while in Aubert et al. [23] experiment was 60 times higher. We found differences between cultivars of the same species for the rutin content. The rutin content was higher in cv. LIT 1 compared to 63481. The differences between cultivars in rutin content were confirmed by [7,22] but not by [23]. The differences in flavonoid accumulation in tartary vs. common buckwheat, rutin in particular, can be explained by the differences in gene expression of genes involved in the flavonoid synthesis pathway. Han et al. and Li et al. [24,25] have shown that genes such as PAL, C4H, 4CL, CHI, FLS, F3H, and F3′H are involved in flavonoid synthesis (including rutin). Multiple copies of these genes were found in the buckwheat genome. This was similarly observed in other plant species. Studies by Gupta et al. [26] found that the PAL, CHS, CHI, and FLS gene expression patterns are dependent on the growth stages and *Fagopyrum* species and cultivars. Interestingly, the transcripts of these genes were more abundant in *F. tataricum* compared to *F. esculentum*. Higher transcript abundance of PAL in the seeds of *F. tataricum* compared to *F. esculentum* was detected and can be directly correlated with high rutin content of *F. tataricum*. In this study, *F. tataricum* contained 43–55 times more rutin compared to *F. esculentum*. Additionally, the CHI gene is known to be an essential gene for the flavonoid biosynthesis pathway and higher CHI expression was found in *F. tataricum* than in *F. esculentum* [27]. Our results and the molecular biology studies suggest that the higher rutin content is a common phenomenon in tartary buckwheat.

Genotype and cultivars influenced the total phenolic acid concentration. The total phenolic acid content of *Fagopyrum tataricum* was higher than that of *Fagopyrum esculentum*. No differences were found in the concentration of total phenolic acids between the tested tartary buckwheat cultivars, which is contrary to research [7]. We found differences in the concentration of total phenolic acids between the tested common buckwheat genotypes. This claim is confirmed by [6,20,28,29,30]. In this study, the vanillic acid was the most prominent phenolic acid in common and tartary buckwheat cultivars. Guo [7] reported that in tartary buckwheat grown in China, p-hydroxybenzoic, ferulic, protocatechuic acid were the prominent phenolic acids, which accounted for 83–88% of the total phenolic acids. In this study, we found that Red Corolla and Green Corolla contain the highest amount of vanillic and syringic acids, while Kora and Panda cultivars contain the highest amount of coumaric acid. 

In this study we measured the concentrations of the nine elements in the buckwheat whole grains samples, and we found significant differences in the Cu, Mn, Fe, Zn, Mg, Ca, Na, K, and P contents of the buckwheat samples. Of the measured minerals, K was the most abundant macroelement in the buckwheat grains, which is confirmed in studies [23,28,31,32,33]. In this study, we found differences between tested genotypes in mineral content. *Fagopyrum tataricum* contains the highest amounts of Cu, Mn, Mg, and Ca compared to *Fagopyrum esculentum*. The Red Corolla and Green Corolla genotypes belonging to *Fagopyrum esculentum* contain significantly more Cu, Mn, Fe, and Zn compared to *Fagopyrum tataricum,* and Kora and Panda cv. The differences between *Fagopyrum tataricum* and *Fagopyrum esculentum* species were found in a previous study. Pongrac et al. [14] reported that, compared to tartary buckwheat, common buckwheat grains contain a larger amount of S, Ca, Cu, and Mo, which is confirmed in this study, but is not confirmed by Aubert et al. [23]. Aubert et al. [23] did not find significant differences between *Fagopyrum esculentum* and *Fagopyrum tataricum* in the concentration of Ca and Cu, but they observed differences between buckwheat species regarding the concentration of Mg, Na, Fe, and Zn. Many factors, including both environmental and genetic influences, can affect the mineral composition of agricultural crops [4,34,35,36]. The different amount of minerals in different buckwheat species may be caused by the differences in the anatomical structure of the seeds and the mutual proportions of the husk to the endosperm and the embryo. Moreover, literature reports that the minerals are distributed in different amounts in the various parts of the buckwheat seeds [4,37].

Buckwheat grains are a rich source of protein with high biological value [5,38]. Generally, the protein concentration of buckwheat grains varies from 12–19%. However, the concentration of proteins varies from species to species and is also influenced by environmental factors, cultivars, and agronomic practices [15,16]. On the basis of qualitative analysis, tartary buckwheat shows a higher concentration of proteins and amino acids than common buckwheat [18,39]. The protein content of tartary buckwheat is 20.2% higher than common buckwheat [17]. It was not confirmed in this study. We found the highest amount of protein in *Fagopyrum esculentum* genotypes. The protein content in Red and Green Corolla genotypes was equal to 14.0%, in Panda cv., 13.7% and in Kora cv., 12.79%. In *Fagopyrum tataricum* seeds it was significantly lower and amounted to 11.5%. Additionally, Aubert et al. [23] reported that seeds of *F. esculentum* contained more proteins (15.3%) than seeds of *F. tataricum* (12.8%). Generally, the buckwheat proteins are composed of albumins, globulins, glutelin, and prolamines [39]. Choi et al. [40] reports that the relative proportions of the protein contents are 21.1%, albumins; 13.8%, globulin; 28.4%, gliadin; and 36.7%, glutelin, whereas [39] reported that the proportion of protein fraction differ in buckwheat species. In *Fagopyrum esculentum,* the protein fraction albumins + globulin, prolamins, glutelins, and residual protein are 38%, 4.5%, 21.08%, and 36.91%, but in *Fagopyrum tataricum* 38%, 1.9%, 28.9%, and 31.0% respectively. This indicates that buckwheat species differ from each other in terms of the proportion of protein fractions. *Fagopyrum tataricum* has more glutelin and less prolamin compared to *Fagopyrum esculentum*. It has been confirmed in this research, however that the proportion of protein fraction was different. Compared to the literature [39,40], we found a higher percentage of albumins + globulin, which was from 51.99% (LIT 1) to 62% (Kora cv.), while a lower percentage of globulin fraction, varying from 10.34% (Kora cv.) to 14.12% (63481 cv.) was found. Moreover, the results obtained in this study for protein fractions in *Fagopyrum tataricum* are in agreement with [41]. They found the albumin to be the predominant protein fraction (43.8%), followed by glutelin (14.6%), prolamin (10.5%), and globulin (7.82%).

## 4. Materials and Methods

### 4.1. Plant Material

A field experiment was established in the years 2017–2018 at the Experimental Station in Osiny (51°35′, 21°55′), Institute of Soil Science and Plant Cultivation–State Research Institute, Pulawy, Poland. The experiment was conducted following randomized complete block method with three replications. Sowing density was set at 250 seeds per 1 m^2^. The area of the harvested plots was 10 m^2^. The buckwheat was sown on pseudopodsolic soil, which is characteristic for the region (winter wheat was the forecrop used), with extractable phosphorus (P: 9.54 mg kg^−1^), exchangeable potassium (K: 12.0 mg·kg^−1^), and pH KCl 6.4. Plots were fertilized with N 40 kg·ha^−1^, P 60 kg·ha^−1^, and K 60 kg·ha^−1^. Sowing terms were 22 May 2017 and 21 May 2018. Mineral fertilization applied at 40 kg N·ha^−1^. Harvest terms were 25 August 2017 and 27 August 2018. No pesticides were applied during the cultivation. Two cultivars of *Fagophyrum esculentum* (Kora and Panda), two cultivars of *Fagopyrum tataricum* (LIT1 cv. and 63181 cv.), and two forms of *Fagopyrum esculentum* (Red Corolla and Green Corolla) were used in this experiment. Red Corolla is a buckwheat line obtained by crossing cv. Hruszowska × cv. Buriatskaja by Joanna Wolinska. It is characterized by red floral envelope and, most importantly, it is more resistant to low temperatures. Green Corolla is a line characterized by green floral and thin husk, whereas Kora cv. is a buckwheat cultivar registered in 1993. This cultivar is characterized by medium-early, good resistance to spring cold and periodic drought. Seeds are quite resistant to scattering. Another cultivar, Panda, was obtained in 1998. It is a medium-early variety with good resistance to spring cold and drought during flowering phase. Seeds of Panda are more resistant to scattering than the Kora variety. The seeds of 63184 cv. and LIT1 cv. were obtained from The National Centre for Plant Genetic Resources. The country of origin for 63184 cv. is Poland and Lithuania for LIT1.

There was no frost after sowing and the weather conditions in 2016 and 2017 during growing season were favorable for the sprouting, growth, and flowering of buckwheat (Table 4).

The whole buckwheat grains were crushed in IKA A10 laboratory mill. The results were calculated as g/100 g of dry matter (d.m.).

### 4.2. Reagents and Chemicals

Standards of gallic acid, rutin, phenolic acids (ferulic, coumaric, syringic, and vanillic), and α-*amylase* were purchased from Sigma-Aldrich (St. Louis, MO, USA). All reagents and solvents used were of analytical or HPLC grade purity.

### 4.3. Total Phenolic Compounds

Spectrophotometry according to the Ribereau-Gayon method in the modification by Guo et al. [7,42] was used to determine the total phenolics compounds. Content of the total phenolic compounds was expressed as gallic acid (equivalent (GAEq.) in µg·g^−1^ of dry matter.

### 4.4. Phenolic Acids

According to the method developed by Pussayanawin and Wetzel [43], some of phenolic acids such as ferulic, coumaric, syringic, and vanillic were determined. These acids were released from buckwheat samples with acid and enzymatic hydrolysis, and then separated with the HPLC method. The samples (2 g) of each species and cultivars were combined with 35 mL 0.1 M H_2_SO_4_ and put into a boiling water for 30 min. Hydrolysis was ended by cooling the samples in an icy water for 10 min before the addition of 5 mL of 2% (*w*/*v*) suspension of α-amylase from Aspergillus oryzae in a 2.5 M aqueous sodium acetate solution. Before being centrifuged at 3000× *g* for 15 min (MPW-350R, MPW Med. Instruments, Warsaw, Poland) the samples were incubated at 30 °C for 1 h. The supernatant was decanted into a 50-mL volumetric flask, followed by its dilution to volume with distilled water. The samples were filtered through a 0.45 μm nylon filter before being injected into the chromatograph. Unit the time of analysis the extracts were protected against UV light and then stored in the refrigerator. The separation conditions applied were the following: Agilent Technologies liquid chromatography (1200 Series); column Phenomenex Synergi RP C18 (250 × 4.6 mm); mobile phase: a solution of 12% methanol in a sodium citrate buffer (pH = 5.4) with isocratic elution; stationary phase: silica gel of 5 μm granulation that was chemically altered with bond aliphatic hydrocarbons with 18 molecules of carbon in a chain; flow rate: 1 mL min^−1^; UV-VIS detector. Ferulic acid was detected at 320 nm, while coumaric, syringic, and vanillic acids were detected at 280 nm. Peak identification was based on the retention time by comparison with standard compounds.

### 4.5. Rutin Content

HPLC method was used to determine the content of rutin [44]. The samples (0.5 g) were extracted with 70% methanol (9 mL) in a shaking water bath at 70 °C for 120 min. After being centrifuged at 3000 rpm for 5 min, the supernatant was taken out and the residue was cleansed with 80% methanol (1 mL). The buckwheat samples were recentrifuged, and the volume of bulked supernatants was made to 10 mL with 70% ethanol. The extracts from buckwheat samples were filtered through a 0.45 µm Millipore Teflon filter, and then separated using Agilent Technologies 1200 Series liquid chromatography with UV-VIS (DAD) detector and column Phenomenex Synergi RP C18 (250 × 4.6 mm). As a mobile phase, a mixture of 2.5% acetic acid, methanol, and acetonitrile was applied in a ratio of 35:5:10 at a constant flow rate (1 mL min^−1^) with isocratic elution. The detection was carried out at 360 nm, and the results were interpreted by the comparison of the peaks of the analyzed samples against the standard separation.

### 4.6. Minerals

The samples of different buckwheat species and cultivars (2 g) were wet-ashed in a mixture of nitric and perchloric acids (20 mL; 3:1; Suprapure, Merck, Darmstadt, Germany) on an aluminum electric heating block (VELP, Milano, Italy) which has a programmable temperature setting. The temperature was gradually increased to 200 °C, which lasted for 2 h. The colorless mineralizate was put to 50 mL volumetric flasks, with deionized water being added for marking purposes. Individual minerals: magnesium, manganese, zinc, copper, iron, and calcium were determined using flame atomic absorption spectrometry (acetylene—air flame) with the iCE 3000 Series Atomic Absorption Spectrometer (Thermo-Scientific, Waltham, MA, USA) with a Glite data station, background correction (deuterium lamp), as well as appropriate cathode lamps [Whiteside, Miner 1984]. The determination of the selected elements was performed at the following wavelengths (nm): Mg-285.2, Mn-279.5, Zn-213.9, Cu-324.8, Fe-248.3, and Ca-422.7. The analyses of potassium and sodium were done using with atomic emission spectrometry (AES), while of P using colorimetric methods (610 nm) [45].

### 4.7. Protein Fractionation

Using the Kjeldahl method (%N × 6.25) [AACC method No. 46-13, 1983, 8th ed. The Association, St. Paul, MN, USA] the protein contents were determined. According to the procedure by Sathe and Salunke [46], the albumins and globulins were fractionated. The solvent for the extraction of prolamins and glutelins was composed according to the modified Osborne procedure by Chen and Bushuk [47].

### 4.8. Statistical Analysis

The randomized block design in three replications was used in this experiment. The data obtained from the field experiments as well as the laboratory tests were statistically analyzed with the use of Statistica v.7.1 software. The analysis of variance (ANOVA) method was chosen to analyze the influence of independent factors (variables). The Tukey’s range test compared the differences between mean values. Additionally, the multifactor ANOVA was run at a significance level of α = 0.05; *—α < 0.05, ns—not significantly different.

## 5. Conclusions

In this study, the genotypes were the main factor that significantly affected the buckwheat quality. In the tested genotypes, total phenolic acid and rutin content were both prominent in the tartary buckwheat cultivars. Furthermore, the ferulic and coumaric acids were prominent in the common buckwheat seed, however vanillic and synergic acids accumulated more in Green Corolla and Red Corolla. The common buckwheat seeds contained more Cu, Mn, and Mg and less Ca than tartary buckwheat. The biggest amounts of this nutrient were found in Red Corolla and Green Corolla genotypes. Due to such high content of those important minerals, Green Corolla and Red Corolla cultivars appear to be excellent candidates that can be implemented in breeding research when high mineral content is the intended feature. This study suggests that tartary buckwheat compared to common buckwheat cultivated in Polish conditions has a significant potential to provide health benefits because of its high total phenolic and rutin content. In contrast, *Fagopytum esculentum* contains more protein compared to *Fagopyrum tataricum*. Differences in tested components (protein, phenolic acids, rutin, and mineral composition) between cultivars obtained in this study indicate that cultivars may contribute more to bioactive properties than species. The scope of the results from this study presents preliminary insights into how varieties grown in Poland’s environment may influence individual buckwheat bioactive compounds. Nonetheless, more in-depth studies with greater number of cultivars belonging to different genotypes are required to better understand these complex relationships.

## Figures and Tables

**Table 1:** The phenolic acid content of buckwheat genotypes (mg·kg^−1^ d.w).

Genotypes						
Traits	*Fagopyrum esculentum*				*Fagopyrum tataricum*	
	Kora	Panda	Red Corolla	Green Corolla	LIT1	63481
Total phenolic acid	2222.2 ± 64.15 ^c^	2856.4 ± 82.46 ^c^	3891.1 ± 112.33 ^b^	2322.7 ± 67.05 ^c^	7014.8 ± 202.50 ^a^	6948.9 ± 200.60 ^a^
Rutin	91.90 ± 2.66 ^d^	114.6 ± 3.29 ^cd^	318.9 ± 9.21 ^c^	143.4 ± 4.13 ^cd^	3257.0 ± 92.96 ^a^	2064.1 ± 59.76 ^b^
Ferulic acid	4.000 ± 0.12 ^a^	3.266 ± 0.09 ^b^	2.696 ± 0.08 ^c^	2.266 ± 0.07 ^d^	2.366 ± 0.07 ^cd^	3.496 ± 0.10 ^b^
Coumaric acid	28.19 ± 0.8 ^b^	39.45 ± 1.14 ^b^	20.49 ± 0.59 ^c^	26.72 ± 0.77 ^b^	15.46 ± 0.45 ^d^	18.81 ± 0.54 ^cd^
Syringic acid	72.00 ± 2.08 ^c^	74.06 ± 2.14 ^b^	85.62 ± 2.47 ^a^	79.94 ± 2.31 ^ab^	38.61 ± 1.12 ^d^	44.34 ± 1.28 ^d^
Vanillic acid	240.0 ± 6.93 ^bc^	254.4 ± 7.22 ^b^	378.0 ± 43.18 ^a^	370.0 ± 10.68 ^a^	155.8 ± 4.61 ^c^	186.6 ± 5.49 ^bc^

Within each row, means with the same letter are not significantly different (*p* < 0.05).

**Table 2:** The mineral content of buckwheat genotypes (mg·kg^−1^).

Genotype						
Traits	*Fagopyrum esculentum*				*Fagopyrum tataricum*	
	Kora	Panda	Red Corolla	Green Corolla	LIT1	63481
Cu	6.39 ± 0.18 ^bc^	7.30 ± 0.21 ^ab^	7.760 ± 0.22 ^a^	7.480 ± 0.21 ^a^	5.94 ± 0.17 ^c^	6.17 ± 0.18 ^c^
Mn	9.373 ± 0.27 ^bc^	8.90 ± 0.25 ^bc^	10.99 ± 0.32 ^a^	9.906 ± 0.29 ^ab^	9.103 ± 0.26 ^bc^	8.160 ± 0.24 ^c^
Fe	24.22 ± 0.7 ^c^	26.38 ± 0.76 ^b^	29.99 ± 0.87 ^a^	30.12 ± 0.87 ^a^	28.17 ± 0.81 ^ab^	25.46 ± 0.73 ^cb^
Zn	47.65 ± 1.38 ^bc^	54.27 ± 1.56 ^b^	63.07 ± 1.82 ^a^	62.68 ± 1.81 ^a^	49.32 ± 1.42 ^b^	40.95 ± 1.18 ^c^
Mg	1445 ± 41.86 ^ab^	1367 ± 39.55 ^c^	1543 ± 44.74 ^a^	1350 ± 38.97 ^bc^	1212 ± 34.93 ^c^	1251 ± 36.08 ^c^
Ca	782.6 ± 22.81 ^c^	772.7 ± 22.23 ^c^	1139 ± 32.62 ^b^	784.6 ± 22.81 ^c^	1413 ± 40.7 ^a^	1176 ± 33.78 ^b^
Na	16.74 ± 0.48 ^b^	12.40 ± 0.36 ^c^	11.77 ± 0.33 ^c^	6.713 ± 1.07 ^d^	14.16 ± 0.41 ^bc^	21.46 ± 0.62 ^a^
K	5939 ± 171.18 ^ab^	5157 ± 148.67 ^b^	6228 ± 179.84 ^a^	5428 ± 156.46 ^ab^	6058 ± 183.66 ^a^	6180 ± 178.4 ^a^
P	3651 ± 105.37 ^b^	3643 ± 105.37 ^b^	3861 ± 111.43 ^a^	3651 ± 105.37 ^b^	3661 ± 105.65 ^b^	3831 ± 110.56 ^a^

Within each row, means with the same letter are not significantly different (*p* < 0.05).

**Table 3:** Mean values of protein content and distribution of protein fractions in the studied buckwheat genotypes (g/100 g d.m.).

Genotype						
Traits	*Fagopyrum esculentum*				*Fagopyrum tataricum*	
	Kora	Panda	Red Corolla	Green Corolla	LIT1	63481
Protein (N% × 6.25)	12.79 ± 0.37 ^b^	13.77 ± 0.4	14.04 ± 0.4 ^a^	14.01 ± 0.4 ^a^	11.48 ± 0.33	11.65 ± 0.37 ^c^
Albumin	7.48 ± 0.21 ^a^	7.00 ± 0.20 ^c^	7.58 ± 0.21 ^b^	6.99 ± 0.20 ^d^	5,68 ± 0.16	5.90 ± 0.17 ^c^
Globulin	0.45 ± 0.01 ^b^	0.32 ± 0.01 ^c^	0.30 ± 0.01 ^c^	0.84 ± 0.02 ^a^	0.29 ± 0.01 ^c^	0.41 ± 0.01 ^b^
Prolamin	0.59 ± 0.02 ^c^	0.81 ± 0.02 ^a^	0.49 ± 0.01 ^c^	0.53 ± 0.01 ^bc^	0.45 ± 0.01 ^bc^	0.48 ± 0.01 ^b^
Glutelin	1.32 ± 0.04	1.67 ± 0.05 ^b^	1.50 ± 0.04 ^c^	1.65 ± 0.05 ^b^	1.59 ± 0.04 ^a^	1.64 ± 0.05 ^a^
Residual protein	2.95 ± 0.08	3.95 ± 0.11	4.17 ± 0.12	4.00 ± 0.11	3.48 ± 0.10	3.21 ± 0.09

Within each row, means with the same letter are not significantly different (*p* < 0.05).

**Table 4:** Rainfall and temperature for the experimental site for the growing period (2016, 2017).

Month	Precipitation mm		Temperature °C	
	2016	2017	2016	2017
May	72.2	48.2	15.6	14.3
June	27.9	56.1	19.8	18.8
July	86.6	56.6	20.1	20.0
August	46.8	60.0	19.7	19.7

## Data Availability

The data presented in this study are available on request from the corresponding author.
